# What Policies Do Local Governments Use to Promote Physical Activity? A Comparative Analysis of Municipalities From 4 EU Countries and Japan

**DOI:** 10.34172/ijhpm.8594

**Published:** 2025-01-27

**Authors:** Sven Messing, Antoine Noël Racine, Noriko Takeda, Tanja Onatsu, Katariina Tuunanen, Antonia Papiu, Leonie Birkholz, Jean-Marie Garbarino, Yuko Oguma, Yoshinobu Saito, Dan Mocan, Răzvan Mircea Cherecheș, Anne Vuillemin, Peter Gelius, Petru Sandu

**Affiliations:** ^1^Department of Sport Science and Sport, Friedrich-Alexander-Universität Erlangen-Nürnberg, Erlangen, Germany.; ^2^Physical Activity for Health Research Centre, Health Research Institute, Department of Physical Education and Sport Sciences, University of Limerick, Limerick, Ireland.; ^3^Université Côte d’Azur, LAMHESS, Nice, France.; ^4^Center for Promotion of Higher Education, Kogakuin University, Tokyo, Japan.; ^5^Adults on the Move Program, Jamk University of Applied Sciences, Jyväskylä, Finland.; ^6^Department of Public Health, Faculty of Political, Administrative and Communication Sciences, Babeş-Bolyai University, Cluj-Napoca, Romania.; ^7^Sports Medicine Research Center, Keio University, Yokohama, Japan.; ^8^Faculty of Sport Management, Nippon Sport Science University, Yokohama, Japan.; ^9^Independent Researcher, Cluj-Napoca, Romania.; ^10^Institute of Sport Sciences, Université de Lausanne, Lausanne, Switzerland.

**Keywords:** Public Policy, Physical Activity, Local Level, Comparative Study, Europe, Japan

## Abstract

**Background:**

As public policies have the potential to change the entire system of physical activity (PA) promotion and to create conducive environments, they are particularly relevant to address the persistently low levels of PA across the world. Furthermore, World Health Organization’s (WHO’s) Global Action Plan on Physical Activity highlights the relevance of local governments as important partners for policy action. However, our knowledge on how local PA promotion policy compares across countries remains limited.

**Methods:**

We conducted an exploratory study as part of the LoGoPAS project to compare the status quo of local PA policies across five municipalities in five different countries. Using purposive sampling, Jyväskylä (Finland), Nice (France), Erlangen (Germany), Fujisawa (Japan), and Cluj-Napoca (Romania) were selected. Data were collected and analysed via desk research and expert consultation using the CAPLA-Santé, a validated tool designed to assess relevant aspects of local PA promotion policies.

**Results:**

The analysis showed that the main responsibility for PA promotion varied between municipalities, resting either with the sport or the health sector. A total of 50 relevant PA policy documents were identified, focusing on multiple settings and target groups. Budgets for PA promotion differed across municipalities. Research on PA was reported to have informed policy development in some but not all cases. Across countries, political support was identified as a key driver of local PA promotion.

**Conclusion:**

LoGoPAS is the first study to apply the CAPLA-Santé outside of France and the first to use it for an international comparative analysis. Results highlight the ability of the tool to provide insights into local PA policy development, contents, and implementation worldwide. While this study provided a cross-sectional in-depth analysis of the status quo in select municipalities, future research could also aim to assess policies at a large scale, ie, for multiple municipalities and/or on a regular basis.

## Background

**Key Messages**

**Implications for policy makers**

- This study can support local governments in further developing physical activity (PA) policies to enhance population health. Leadership for PA promotion can be taken by the municipal sports or health department, or by institutions combining competencies of both sectors.
- Policy documents for PA promotion can target different sectors individually (eg, sports, transport, health, environment, social, urban planning, education, and tourism) or address multiple sectors simultaneously (eg, via city development strategies).
- Policies should include objectives for increasing PA, targeting different settings and populations. They may be implemented through programmes and interventions.
- Funding specifically dedicated to the promotion of PA is needed, as are studies at local level to inform policy development.
- In the municipalities studied, the current status of PA promotion was influenced by the municipality’s organisational structure, political support as well as existing policy documents and projects.

**Implications for the public**

Physical activity (PA) has numerous health benefits. However, studies have shown that a large percentage of the population is not as active as they should be. To promote health in the population, policies at the local level are important. Such policies can take local characteristics into consideration, implement national and regional policies and/or initiate policy development (rather than waiting for higher levels of government to take action). Political support is a key driver of local PA policy development, and could be strengthened by civil society advocacy on the importance of policy development, implementation, and adequate funding mechanisms. However, promoting PA at the local level may vary greatly depending on the local context, institutional setup, and politics. Therefore, individually-tailored solutions need to be found.

Physical inactivity is a global problem, as 27.5% of adults and 81.0% of adolescents do not reach recommended physical activity (PA) levels.^1,2^ The COVID-19 pandemic seems to have worsened the situation further, with worldwide step counts having been significantly lower for at least two years compared to pre-pandemic levels.^3^ Estimates indicate that insufficient PA is responsible for 9% of premature mortality, 10% of breast cancer and colon cancer, 7% of type 2 diabetes, and 6% of coronary heart disease.^4^ Consequently, it is highly important from a public health perspective to reduce the level of inactivity. Policy approaches are of particular relevance as they have the potential to change the entire system of PA promotion and to create conducive environments,^5,6^ and there is evidence for the impact of policy on PA outcomes within a number of different settings.^7-11^ Policy is defined as formal or informal legislative or regulatory action, statements of intent, or guides to action issued by governments or organizations.^12-15^ In particular, “policies are not individual measures or actions to promote PA – they are not interventions but the framework in which interventions are tendered, developed, financed, or implemented.”^11^

 As early as 2006, the World Health Organization (WHO) stated that “local governments have a crucial role to play in creating environments and opportunities for PA and active living.”^16^ In 2018, WHO set the goal to reduce physical inactivity by 15% until 2030,^17^ and explicitly recommended working together with “city leaders and local government” to achieve this.^17^ These statements have been reinforced by scientists who called to plan globally and act locally for PA: In 2021, Pratt and colleagues stated that “a cascade from global to national to local levels is a critical missing piece” in PA promotion and proposed a closer investigation of policies and their implementation at the local level.^18^ The authors argued that cross-sector collaboration—which is highly important to promote PA effectively—is less difficult and therefore more common in municipalities than at national level.^18^ This allows for the development of cross-sectoral strategies addressing the “investments that work for PA,” such as whole-of-school programmes, the promotion of active transport, and sport and recreation for all.^19,20^ From our perspective, it seems to be important to take action at the local level (*a*) to take local characteristics into consideration, eg, differences between cities and rural areas, (*b*) to implement national and regional policies, and/or (*c*) to develop local policies rather than waiting for higher levels of government to take action. This is in line with previous studies that discussed some of these aspects.^21-24^

 However, research on PA policy development and implementation focuses mainly on the national level. A potential explanation for this focus is that most countries adopted formal written PA policies at national level.^25^ These policies are analysed in scientific studies, and a recently published review on the monitoring and assessment of PA policies across different levels of government showed that 85 out of 112 studies focused on the national level.^26^ Several of these studies used highly visible tools to monitor or assess PA policies, such as the European Union’s (EU’s) PA Country Factsheets,^27^ WHO’s Health-Enhancing Physical Activity Policy Audit Tool (HEPA-PAT),^28^ the GoPA! country cards,^29^ and the newly developed PA Environment Policy Index.^5^ In contrast, only five (out of 112) studies focused on the local level, and only a very limited number of tools specifically designed for the local level is available.^26^ Examples include the CAPLA-Santé, the Active Community Environments tool and the Community Health Assessment and Group Evaluation tool.^30-32^ Furthermore, the INTEGRATE-PA-Pol tool has recently been developed to assess PA policy development at national and local level.^23^

 The research gap on local PA policies is confirmed by another review stating that little is known about local evidence to advise and support governments in their policies to promote PA.^21^ To date, only a few studies have analysed local PA policies in selected municipalities in the United States,^30,31^ France,^33^ and Latin America.^24^ However, these studies are based on a heterogeneous methodology which makes it difficult to compare their findings systematically.

 This study aims to compare the status quo of local PA policies across five municipalities from five different countries. To the best of our knowledge, this is one of the first studies to compare local PA policies across different countries based on a common methodology.^24^ This comparative approach may be an added value from a methodological perspective, as it can inform the future use of tools to assess PA policies. In addition, selected results may serve as a reference for other municipalities by providing good practice examples for PA promotion at the local level.

## Methods

###  Study Design

 This exploratory study was conducted in the five municipalities Jyväskylä (Finland), Nice (France), Erlangen (Germany), Fujisawa (Japan), Cluj-Napoca (Romania) as part of the EU-funded project “LoGoPAS – Assess, promote and support the involvement of local governments in PA and sport promotion.”^34^ All five municipalities are located in very highly developed countries according to the Human Development Index; however, while Romania is ranked 53rd, the other countries are in the top 30 (Japan, France) or even in the top 15 (Germany, Finland).^35^ Also the gross domestic product (GDP) per capita is the lowest in Romania (around US$ 18 000), and considerably higher in Japan (US$ 34 000), France (US$ 44 000), Germany (US$ 53 000), and Finland (US$ 54 000). According to The Economist’s Democracy Index, France, Finland, Germany, and Japan can be considered “full democracies,” while Romania is described as a “flawed democracy,” ie, a country with free and fair elections where basic civil liberties are respected but which has significant weaknesses in aspects such as governance, political culture and/or political participation.^36^

 The municipalities were selected using purposive sampling based on the following guidelines agreed upon by the project consortium: (*a*) case examples were to represent different regions in Europe (Northern, Western, and Central and Eastern Europe) and a comparative case outside Europe (Japan); (*b*) municipalities were supposed to have a roughly comparable size with populations between 100 000 and 500 000; and (*c*) case examples were supposed to have a high level of prior engagement in PA promotion and excellent potential for collaboration with the researchers in each country. The latter criterion was paramount as the project aimed to collect and showcase examples of “active” municipalities and of good PA promotion practice, and to engage local policy-makers in discussions with each other and with researchers. In addition to this study, policy-makers were also engaged in the development and deployment of local meetings (two in each of the five municipalities, throughout the project development) and one transnational meeting, held in Nice (France) as part of the project implementation plan.

###  Data Collection

 Data were collected using the CAPLA-Santé, a tool designed to support local governments in assessing policies for PA promotion.^37^ The CAPLA-Santé is a local-level adaptation of WHO’s HEPA-PAT,^28^ a standardized instrument originally designed to assess national-level policies for PA promotion. The CAPLA-Santé contains 21 items in six sections covering aspects such as stakeholders and policy documents relevant for PA promotion, as well as the availability of funding.^32^ At the time of the LoGoPAS study, only French and English versions of the tool were available; to facilitate data collection and verification, the CAPLA-Santé was translated into Finnish, German, Japanese, and Romanian.

 Data in the five municipalities were collected in a three-step process between 2020 and 2022. The process of data collection and analysis was modelled on an earlier international comparative study of national-level policies based on the HEPA-PAT.^38^

 In a first step, the international project coordination team divided the questions of the CAPLA-Santé into two categories: those that could be addressed by conducting desk research (eg, describing the public institutions responsible for PA policies), and those that required expert consultation (eg, opinion on local success factors). Adaptations were made by each national partner depending on the specific context and data availability in each municipality.

 In a second step, questions that could be addressed by conducting desk research were answered. This included the identification of policy documents (eg, local PA strategies or action plans), city council decisions, documentation of approved or completed projects, or local media reports that could support or complement information from official sources.

 In a third step, 59 policy-makers and stakeholders were involved in the data collection and verification process. The methodology for involving these groups of experts differed between the municipalities in order to take into account the respective local context. In total, 15 expert interviews were conducted (11 group interviews in Fujisawa, Japan, with representatives of 11 municipal departments involving 26 city officials; and one expert interview with one city official in each of the four other municipalities). In addition, stakeholder workshops (Cluj-Napoca and Jyväskylä) or e-mail feedback loops (Erlangen, Nice, and Fujisawa) were used to obtain feedback on a preliminary CAPLA-Santé draft. The policy-makers involved were municipal employees in various positions, including heads of departments and program managers. Stakeholders representing civil society attended the workshops in Cluj-Napoca and Jyväskylä (eg, representatives of nongovernmental organisations from different sectors).

###  Data Analysis

 Data analysis aimed to map PA policy at the local level and to identify similarities and differences between the five municipalities. Drawing on previous studies of cross-national comparisons of national PA policies based on the HEPA-PAT,^38-40^ the data were analysed by using directed content analysis, ie, the sections of the CAPLA-Santé served as initial themes for the analysis. As the answers to the CAPLA-Santé questions represent the consolidated information from the previous steps—ie, desk research and the expert opinion of individuals and organisations involved—they were not coded in the way usually employed for qualitative interviews. Instead, initial results were identified in a first screening of all five CAPLA-Santés, fed back to the LoGoPAS researchers for comments and clarification, and discussed to ensure that key results are complete and accurate.

 For reporting the results, data were structured according to the six sections of the CAPLA-Santé: (1) HEPA stakeholders, (2) policy documents, (3) policy content, (4) funding and political engagement, (5) studies and measures relating to PA in the local government area, and (6) progress achieved and future challenges.

## Results

###  HEPA Stakeholders (Section 1)

 The responsibility of public organisations from different sectors for PA policies differed across municipalities (*question 1*). While the municipal sport departments are the *key institutions* in Jyväskylä (Finland) and Cluj-Napoca (Romania), the municipality health department has the leadership for PA promotion in Fujisawa (Japan) and in Nice (France). In Erlangen (Germany), an Office of Sports and Health Promotion is in charge, ie, an organisation that was originally responsible for sport only but whose competences were recently expanded to include PA and health promotion. In all municipalities, the importance of other sectors and of shared responsibility for specific aspects of PA promotion was recognized. Besides sport and health, the departments in charge of transport/infrastructure, finances, education, youth, senior citizens, social services, planning/building, tourism, and environmental policies were described as responsible public organisations in the field of PA promotion.

 Across municipalities, a range of *non-governmental stakeholders* are actively engaged in HEPA promotion (*question 2*). While respondents from four municipalities stated that organisations from both the sport and health sector are actively engaged (such as sport clubs and health associations), Cluj-Napoca (Romania) reported stakeholder engagement mainly for the sport sector. In all cases, academic institutions are engaged in HEPA promotion, eg, a university hospital centre, a school of public health or a faculty of physical education and sport.

 Different organizations and key actors were *driving forces for PA promotion* (*question 3*). While the French and German municipalities highlighted the key role of non-governmental organizations (especially sports clubs), the municipalities in Japan and Romania stressed the importance of leadership by local administrations, responsible departments within these administrations, and/or a political champion (ie, in Cluj-Napoca, Romania). In Jyväskylä (Finland), companies and commercial operators were perceived as additional key players.

 Even though *cross-sectoral collaboration* occurs in all municipalities, the extent to which certain organizations formally coordinate the development and implementation of HEPA policies differs (*question 4*). This is illustrated by two examples: While Erlangen (Germany) reported numerous formal processes that facilitate cross-sectoral collaboration within the city administration, formalized collaborations in Cluj-Napoca (Romania) only take place in the context of specific events or projects and are symbolic rather than instrumental, as only the lead stakeholder is held responsible for the successful implementation of the action. For example, when the municipality is assigned a partnership position in privately-developed PA-related programs or events, its role is often limited to formally approving them (eg, providing permits for public spaces, renting out public facilities at a discount or free-of-charge to the organisers).

 Similarly, the form of *networks* to implement HEPA policies (*question 5*) varies widely across municipalities. While the municipalities in France and Japan reported the existence of a single network that has a key role in PA promotion (Azur Sport France, Fujisawa City Health Promotion Meeting), the municipality in Germany named various networks each focused on a specific setting (eg, sport, health, and worksite) and/or target group (eg, people with disabilities, refugees). Networks in Cluj-Napoca (Romania) are often informal and formed around individuals who have collaborated with each other in the past. As these informal networks are not backed by formal collaborations between organizations/sectors, contacts and collaboration opportunities often cease when a person leaves.

###  Policy Documents (Section 2)

 All in all, 50 *policy documents* which indicate the local government authorities’ intentions to promote PA (*question 6*) were identified across the five municipalities. This includes both multi-sectoral policies as well as policies from specific sectors such as sport, transport or health (Table 1).

**Table 1:** Policy Documents

	**No. of Policy Documents**	**Examples of Identified Policy Documents (Release Date)**
Multi-sectoral	9	Finland: Jyväskylä City Strategy 2017-2020 (2017) Finland: Plan for well-being 2017-2020 (2017) Romania: Cluj-Napoca Development Strategy 2014-2020 (2014)
Sport sector	11	Germany: Guidelines for Municipal Sports Promotion (2020) Romania: Reglementations for financing sport programs (2020)
Transport sector	8	France: Metropolitan Bicycle Action Plan 2021-2026 (2021) Romania: Approval of extending the cycling infrastructure (2020)
Health sector	7	France: Physical activity program “Nice Acti’Health” (2019) Germany: Collaborative health strategy of the Health Region^plus^ (2017)
Environment sector	5	Finland: Viherpalveluohjelma KymppiV (Green services programme) (2020)
Social sector	4	Japan: Health and Welfare Plan for the Elderly (2021)
Urban planning sector	3	Finland: OUR Urban Environment policy (2020) Germany: Socially Integrative City – Study Area (2019)
Education sector	2	Japan: Lifelong Learning Fujisawa Plan 2021 (2017)
Tourism sector	1	Japan: Fujisawa City Tourism Promotion Plan (2011)

 All municipalities confirmed that the identified *policies complement each other* (*question 7*). However, while Fujisawa (Japan) stated that each policy is developed based on a single policy document (“Comprehensive Guidelines for Fujisawa City Policies”), the other municipalities stated that policies tend to remain separate, that there is little cross-sectoral collaboration and that several different basic policy documents provide a point of reference for all further action. Some municipalities reported that efforts were made to integrate different sets of policy recommendations (Germany) and to link policies across different sectors and to municipal budget preparations (Finland).

 Finland, France, and Romania reported to have *policy documents based on scientific evidence* (*question 8*), such as reviews of the academic literature or the results of national and/or local studies. Erlangen (Germany) stated that projects are often based on scientific methods of knowledge co-production. Fujisawa (Japan) highlighted the importance of national or regional policy documents as a basis for local policies besides scientific evidence.

###  Policy Content (Section 3)

 All municipalities identified *objectives* for increasing PA (*question 9*). These focused on PA behaviour in general (share of the population that is physically active), active transport (eg, to increase bicycle use), health and well-being (eg, to achieve the highest healthy life expectancy in the country), sport (eg, to have the most active residents in the country), or the structures for PA promotion (eg, to facilitate collaboration between stakeholders).

 The number of *settings* targeted by the development of HEPA actions (*question 10*) varied widely across the investigated municipalities (Table 2). While Jyväskylä (Finland) targets almost all settings that are included in the CAPLA-Santé, the municipalities in France, Germany and Romania pooled their resources for actions in selected settings. All municipalities targeted the urban environment as well as sport and leisure. In contrast, no municipality developed HEPA actions targeting the prison environment (possibly due to the responsibility of higher levels of government), and only one municipality focused on the rural environment or nurseries and infant schools.

**Table 2:** Settings Concerned by the Development of HEPA Actions

	**Finland (Jyväskylä)**	**France (Nice)**	**Germany (Erlangen)**	**Japan (Fujisawa)**	**Romania (Cluj-Napoca)**
Urban environment	X	X	X	X	X
Rural environment	X				
Work environment	X		X	X	X
Prison environment					
Nurseries and infant schools	X				
Primary school	X	X			X
Secondary school	X				X
University	X			X	X
Health centres, nursing homes	X			X	
Health and social care centres	X			X	
At home	X			X	
Sport leisure	X	X	X	X	X
Transport	X	X		X	X
Tourism	X			X	
Environment	X	X	X	X	
Urbanism	X	X		X	

Abbreviation: HEPA, Health-Enhancing Physical Activity. X = Setting targeted by HEPA actions in the respective municipality.

 All municipalities addressed a broad range of *target groups* (*question 11*) with their HEPA actions (Table 3). However, only one or two municipalities reported HEPA actions targeting vulnerable people, workers/employees or migrants. The extent to which municipalities focused on various target groups also differed. For instance, Jyväskylä (Finland) reported that although all target groups are addressed in some way, services are slightly more focused on the earlier stages of life.

**Table 3:** Target Audiences of HEPA Actions

	**Finland (Jyväskylä)**	**France (Nice)**	**Germany (Erlangen)**	**Japan (Fujisawa)**	**Romania (Cluj-Napoca)**
Pre-school	X		X	X	X
Children/Adolescents	X	X	X	X	X
Students	X		X	X	X
Women	X		X	X	X
People in care facilities/patients suffering from chronic diseases	X	X		X	
General population	X	X	X	X	X
Sedentary people	X	X	X		X
Inactive people	X	X	X	X	X
Vulnerable people	X				
Adults	X	X	X	X	X
Families	X	X	X	X	X
Working/employees	X			X	
Migrants	X		X		
Disabled individuals	X	X	X		X
Seniors	X	X	X	X	X

Abbreviation: HEPA, Health-Enhancing Physical Activity. X = Target audiences of HEPA actions in the respective municipality.

 All municipalities used *recommendations* on PA and/or sedentary behaviour (*question 12*) as a basis for their policy-making. While the municipalities in Finland, France, Germany, and Japan referred to their respective national PA recommendation,^41-44^ Cluj-Napoca (Romania) used an adapted version of the WHO PA guidelines, inspired by a national level campaign. Erlangen (Germany) reported thatcountry-specific recommendations and guidelines published by different organizations—such as the National Association of Statutory Health Insurance Funds and the Federal Centre for Health Education—also have a high relevance.

 All municipalities used *communication strategies* or actions (*question 13*) to promote PA. Aspects highlighted included the use of different tools and media channels, the targeting of messages, the use of “door openers” (people from the target group with a high communication potential), naming a responsible officer for communication related to sports and PA, and the use of events to promote sport and exercise offers. Jyväskylä (Finland) and Cluj-Napoca (Romania) created videos about their sports and PA actions for dissemination through local media outlets.^45,46^

 In all municipalities, *programmes or interventions* were conducted to implement local PA policies (*question 14*). Examples include projects targeting senior citizens or women in difficult life situations, projects focusing on the promotion of walking, and projects focusing on playgrounds for children or fitness equipment for adults/older adults in parks (Table 4).

**Table 4:** Programmes or Interventions

	**Programme/Intervention**	**Brief Description**
Finland (Jyväskylä)	Jyväskylä – The Capital of Sport	PA promotion (including sporting events and wellness tourism) as one of four strategic points of the city strategy (adopted by the city council).^47^
	Rantaraitti recreational route	Recreational route circling a lake for walkers, cyclists, and inline skaters (including 15 fitness stops and workout stations, Finland’s largest outdoor gym).^48^
France (Nice)	Seniors in shape	PA offer for senior citizens (combined with cultural activities), offered by the Senior Citizens Department.^49^
	Nice Acti’Health	12-week PA offer for adults with NCDs and senior citizens, combined with psychological support (art therapy) and dietary monitoring.^50^
Germany (Erlangen)	BIG (Movement as an Investment for Health)	Demand-oriented sports and PA offers for women in difficult life situations (around 60-70 courses and events per year), developed using a participatory approach.^51,52^
	GESTALT (Walking, Playing and Dancing as Lifelong Activities)	Holistic exercise program for senior citizens to prevent dementia; improvement of physical, cognitive and psychosocial resources.^53,54^
Japan (Fujisawa)	Fujisawa +10 Project	Community-wide approach to promote PA primarily for senior citizens.^55,56^
	Fujisawa Dream Challenge – Fujisawa Walking Project	Promotion of walking through the provision of information on spots to visit, walking maps and the use of apps to measure steps.^57^
Romania (Cluj-Napoca)	Playgrounds and fitness equipment in parks	Promotion of PA in public parks through playgrounds for children and fitness equipment for adults and senior citizens (financed from local budget or EU funding).
	ClujBike	Free-of-charge bike sharing system, developed through EU co-financing and administered from municipal funds (400+ bikes, 40+ stations, 40 000+ registered users).^58^

Abbreviations: PA, physical activity; EU, European Union; NCDs, non-communicable diseases.

###  Funding and Political Engagement (Section 4)

 In each municipality, *funding* specifically allocated for the implementation of PA policies (*question 15*) was available. As funding sources, researchers identified the municipal budget, funding by regional and national governments, sponsorship (eg, for specific projects or sporting events), participation fees, and university research funds (Japan). Due to the involvement of many different political sectors, it was not possible to identify the exact amount of funding dedicated to PA promotion.

 In Finland, the resources directly available to municipalities are comparatively ample, while they are smaller in Germany due to strong financial support of the regional and federal level, eg, for sports infrastructure. The use of EU funding differed across the four European municipalities: While it has been important in Cluj-Napoca (Romania), the other municipalities relied mostly on national, regional and local funding. However, Jyväskylä (Finland) reported to be exploring the use of EU funding to promote future PA promotion initiatives.

###  Studies and Measures Relating to Physical Activity in the Local Government Area (Section 5)

 Local surveys, studies, or measures providing *HEPA metrics* were conducted in Finland, Germany, Japan, and Romania (*question 16*). Data were collected via surveys, interviews, the measurement of physical capabilities, or bicycle counting systems. The studies focused on relevant behaviours (PA, sport, exercise, cycling, walking, sedentary behaviour), determinants (physical capabilities, bicycle traffic safety), outcomes (well-being, health), and organizations (sport clubs). While most studies collected data from the general population, some focused on a specific target group such as children and adolescents, people with disabilities or older people. Most of the studies were conducted on a regular basis (eg, annually or bi-annually) while others were carried out on a one-time basis (eg, in Cluj-Napoca, Romania, within the context of a municipal strategic development plan). In contrast to the other municipalities, no studies on HEPA metrics have been conducted in Nice (France).

 The four municipalities conducting surveys or other studies reported that their results *influenced policy development* (*question 17*). An example is the development of policies that help to overcome barriers of being active: In Fujisawa (Japan), adults reported a “lack of time” and “too much trouble” as key reasons for not being active (Fujisawa city, 2020). To address this issue, the city focused on the promotion of walking, as this behaviour can be integrated easily into everyday life.

 So far, no municipality has conducted a *cost-benefit study* (*question 18*) of its PA policies. However, this has been discussed in Jyväskylä (Finland) as it could help evaluate the effectiveness of local level policies and related investments in future.

###  Progress Achieved and Future Challenges (Section 6)

 All municipalities identified *people, documents, events or moments that played a key role *for policy development (*question 19*). Finland, Germany and Japan stated that organisational aspects such as the foundation of a local authority with the relevant competences played a key role for PA policy development. The administration’s internal structure matters as well: In Jyväskylä (Finland), the municipal sports service was reorganised due to the increasing complexity of its tasks and gained additional responsibilities which resulted in a more diverse expertise of the organisation’s staff (eg, due to a change of their recruitment policy); for these reasons the reorganisation of the municipal sports services was considered highly relevant for subsequent policy development. For Cluj-Napoca (Romania), consultation processes with citizens were significant for the development of the municipal strategy. Additionally, political support (from individual policy entrepreneurs, across political parties, and from the national government), existing policy documents (in connection with human resources to support policy implementation), and projects (also in collaboration with universities) played a key role in different municipalities.

 Across all municipalities, *strengths and weaknesses* of the local governments in the area of PA promotion were identified (*question 20*). Important strengths were political commitment, the institutional environment (eg, support from a national agency or local university), support from the sport sector (high number of members, sports-for-all policies), and support from the health sector (endorsement of policies by a local network of health practitioners). Frequently identified weaknesses were inefficient structures within the local administration and a lack of intersectoral collaboration. In addition, the dependence on national policy in each sector, the limited amount of resources and a lack of expertise within the municipality were described as weaknesses.

 The sectors with the *most progress* in HEPA promotion in recent years were different in each municipality (*question 21*). While Erlangen (Germany) highlighted positive developments in the sport sector, Fujisawa (Japan) singled out the health sector. In Cluj-Napoca (Romania), the transport (cycling infrastructure) and urban planning (fitness equipment in parks) had advanced the most. Nice (France) reported major progress in human resource investment, eg, the recruitment of professionals to implement the city’s HEPA policy.

 The municipalities identified several *challenges* in launching or pursuing actions to promote HEPA (*question 21.b*). Most importantly, behaviour change was considered a long-term process that might conflict with traditions and cultures. Other challenges identified were overcoming problems within local administration structures, effectively involving stakeholders (eg, doctors for PA counselling), reaching socially disadvantaged people, building and maintaining walking infrastructures, and the lack of research related to the local level.

 All municipalities formulated *advice for other local governments* in which HEPA policies do not exist or who are in middle of the development process (*question 21.c*). The results are summarized in Box 1.

**Box 1. Advice to Other Local Governments**

♦ Make investments in long-term developments to achieve gradual but steady improvements.
♦ Make special investments in children and young people while not neglecting adults and senior citizens.
♦ Adapt strategies to different age groups.
♦ Find ways to reimburse PA courses through the social security system.
♦ Improve urban design and collaborations with schools to promote active transport.
♦ Dismantle rigid structures within the local administration and cultivate a culture of interdisciplinary needs assessment.
♦ Increase the visibility of the department responsible for PA within the local administration.
♦ Prioritize the promotion of PA and sports on the policy agenda and ensure political support.
♦ Involve citizens in the development of PA policies or action plans.
♦ Evaluate programs and interventions to objectively quantify their impact.

-------------

Abbreviation: PA, physical activity.

## Discussion

 The LoGoPAS study aimed to compare the status quo of local PA policies across five municipalities from four EU member states and Japan. It used a validated data collection instrument, the CAPLA-Santé, which is divided into six sections. The study indicated that public organisations from the sport or health sector bear a main responsibility for PA promotion, while differences between municipalities might be influenced by the structure of the political-administrative systems (*section 1*). In all municipalities, policy documents relevant for PA promotion could be identified (*section 2*). These local policies focus on multiple settings and target groups (*section 3*). Differences in the available budget were identified, which can be partly explained by the different possibilities for financial support from higher political levels. However, due to the involvement of many different political sectors, it was not possible to determine the exact amount of funding allocated to PA promotion (*section 4*). In four out of five municipalities, PA-related studies have been conducted (eg, surveys), influencing policy development (eg, the municipality strategy in Cluj-Napoca) (*section 5*). Political support was a key driving force for the current progress in PA promotion, but different aspects were highlighted by each municipality (eg, individual policy entrepreneurs, policy documents, or the institutional structure) (*section 6*).

 Our results are in line with previous studies but provide additional insights into local level PA promotion. For instance, municipalities reported that policies tend to remain separate despite efforts to integrate PA promotion efforts across different sectors. Similar results have been reported for the national level in a study analysing PA promotion in four EU member states.^59^ This national-level study showed that effective cross-sector cooperation requires common objectives, a common budget, decision-making mechanism and clearly defined responsibilities.^59^ Likewise, the challenges we identified for PA promotion at local level (eg, structures within the local administration, involvement of stakeholders, building and maintenance of infrastructure) are complementary to another study: Howie & Stevick have found that the main reasons for the failure of school-based PA policy implementation are inadequate capacity, inappropriate measures, and insufficient funding.^60^ Furthermore, we identified similar key driving forces for local PA policy as previous studies, eg, the existence of a responsible department within the local government, political commitment (eg, by a policy entrepreneur), and the development of a city strategy.^61^ Our results also show that different types of evidence are used at the local level, including policy documents developed by higher levels of government. This is in line with a previous study by the REPOPA consortium which identified the use of five types of research evidence in PA policy, including the wider social context (eg, laws, economics, government policy), the media, and everyday knowledge and intuition.^62^ However, even though four out of five municipalities conducted surveys or studies on HEPA metrics, it is striking that none of them has conducted a cost-benefit study. Such studies—and other evaluations of the effectiveness of policies in a specific local context^63^—could stimulate future policy development by municipalities.

 From a more general perspective, this study contributes to addressing the research gap on local PA policies by comparatively analysing PA policy-making in five municipalities from five countries using a standardized data reporting tool. As described by Noël Racine and colleagues, it thus addresses the need “to have a comprehensive overview of the aspects of interest for research and practice” by using a systematic method.^21^ The study contributes to the scientific literature on this topic by presenting detailed insights into local PA policy development and by being, to the best of our knowledge, one of the first international comparisons of local PA policies at this level of detail. The results of this study also suggest that there may be a causal relationship between the national political and economic context and differences in local PA policies: Romania has a significantly lower GDP per capita than the other four countries, and Cluj-Napoca was the only municipality in this study to report the high importance of supranational (EU level) funding for PA promotion. The political-administrative structure within a country also seems to play a role, as a comparison between Jyväskylä and Erlangen shows (two municipalities in countries with an almost equal GDP per capita). While Jyväskylä has a lot of financial resources directly available at the local level, Erlangen can access such resources indirectly through financial support available at the national and regional level. Future international comparisons could further investigate if and how the political and economic context can explain differences but also similarities in local PA policies (eg, whether different types of political support are required in different political contexts). Another interesting perspective is the link of national and subnational level PA policies which has recently been investigated for four Latin American countries.^24^

 The study is also the first to use the CAPLA-Santé tool for an international comparison. The tool was developed in France and has so far been applied to 10 municipalities on the French Riviera.^33^ The application in municipalities in three additional EU countries and Japan can therefore inform the future use of the CAPLA-Santé in other countries, and provides new versions of the tool in four additional languages. For municipalities outside France, minor adjustments of single indicators might be beneficial to adapt the tool to a country-specific context (eg, *question 10* originally included “priority neighbourhoods for urban policy” and “neighbourhoods (other than priority areas)” as additional settings; however, these terms refer to the political-administrative system in France). In addition, the process of applying the tool might have to be adapted to requirements of a municipality, and the methodology described in this study might provide guidance.

 The study showed that the level of policy-maker involvement in applying the CAPLA-Santé can differ across municipalities. A recently-published review of PA policy monitoring tools differentiated between research-driven, government-driven, and co-production approaches.^26^ Within the LoGoPAS project, policy-makers and/or relevant stakeholders were involved in all municipalities. However, the process in the French, German, and Japanese municipalities can be interpreted as a mainly research-driven approach (ie, researchers led the data collection and involved policy-makers mainly for the purpose of additional data collection and data verification), while the process in the Finnish and Romanian municipalities was closer to a co-production approach (ie, researchers and policy-makers had a series of meetings related to data collection, verification, and/or capacity building for PA promotion). Both approaches have their strengths and weaknesses but it has been highlighted in the scientific literature that co-production approaches can “produce research findings that are more likely (…) relevant to and used by the end users.”^64^

 From a broader public health and public policy perspective, an important question is whether and how the CAPLA-Santé can be used to monitor PA policy at a large scale. While the tool facilitates an in-depth analysis of local level policies,^37^ our experience confirms that its open format and co-productive process require ample staff resources and comparatively long timelines. Other local-level PA policy monitoring tools, such as the Finnish TEAviisari as well as the Local Policy Audit Tool and City Policy Audit Tool currently in use in Japan, are questionnaire-based and thus allow for collecting data faster and from much higher numbers of municipalities but yield less information per municipality.^65-67^ In our opinion, a combination of tools (eg, regular questionnaire surveys backed up by in-depth case studies at greater intervals) might be a good compromise, not least as three of them – CAPLA-Santé, Local Policy Audit Tool, and City Policy Audit Tool – are based on WHO’s HEPA PAT, thus facilitating the comparability of results across instruments.

 This exploratory study has several limitations. First, it is based on a small number of municipalities that are not representative for their respective country (high level of prior engagement in PA promotion). Second, within each municipality, only a limited number of policy-makers were involved, thus increasing the risk of bias, as perceptions of PA policy development can vary substantially across sectors and might depend on the formal position of key informants.^61^ Third, even though data collection was guided by a standardized process in all municipalities, minor methodological differences and limitations could not be avoided to cater for contextual differences. In addition, the translation of the tool into the respective national languages might limit the comparability of results across countries. Finally, the LoGoPAS study was an assessment of local PA policies at one point in time, and not integrated into a regular monitoring of PA policies in the selected municipalities (limiting our knowledge on policy development and implementation over a longer period time of time).

## Conclusion

 The LoGoPAS study provides valuable insights into the status quo of local PA policies in five municipalities from different countries. It showed that public organisations, primarily from the sport or health sector, play a key role in promoting PA. The study identified relevant policy documents in eight different sectors, and highlighted the importance of intersectoral collaboration political support, local evidence, and financial resources for the promotion of PA by local governments. The findings also suggest that the availability of funding may depend on the economic context and on the political-administrative structure within each country. However, these hypotheses are based on a small number of cases, and may need to be tested in future studies.

 From a methodological perspective, this is the first international comparison of local PA policies based on the CAPLA-Santé, and the first application of the tool outside France; therefore, the methodology used to apply the tool could provide guidance for future studies. Further case studies are needed to test the tool in additional national (other countries and world regions) and local (rural areas, municipalities with less developed PA policies) contexts. Besides, future research should also aim to assess local PA policies comparatively at a larger scale. Such an assessment should ideally cover all municipalities within a country or region, and take place on a regular basis as part of a monitoring system (as, for example, the Finnish TEAviisari^66^). For additional insights, such large-scale data collections could be combined with in-depth studies based on the CAPLA-Santé or comparable tools. Finally, as most tools for PA policy assessment were developed in specific (often “global north”) political contexts, their universal applicability should be investigated further, especially with regards to low- and middle-income countries.

## Ethical issues

 The work was approved by the members of the Institutional Review Board – Public Health (IRB-PH), University Babes-Bolyai (June 09, 2021; Ref. No. 210609-008).

## Conflicts of interest

 Authors declare that they have no conflicts of interest.
